# Classification Performance of a Deep Learning Algorithm for Common Skin Lesions in the Emergency Department: A Multicenter Preliminary Validation Study

**DOI:** 10.3390/jcm15218602

**Published:** 2026-11-04

**Authors:** Dong Eun Lee, Jong Kun Kim, Min-Seong Kwak, Kyung-Woo Lee, Sin-Youl Park

**Affiliations:** 1 Department of Emergency Medicine, School of Medicine, Kyungpook National University, Daegu 41944, Republic of Korea; reveur2010@naver.com (D.E.L.); kim7155@knu.ac.kr (J.K.K.); 2 Department of Emergency Medicine, College of Medicine, Yeungnam University, Daegu 42415, Republic of Korea; emergency99@naver.com; 3 Department of Emergency Medicine, Daegu Catholic University Hospital, Daegu 42472, Republic of Korea; turtle072@hanmail.net

**Keywords:** deep learning, skin diseases, emergency department, diagnosis, computer-assisted, artificial intelligence, clinical decision support systems

## Abstract

**Background/Objectives**: Initial classification of skin lesions in the emergency department (ED) is difficult when dermatology consultations are limited. This study externally validated a deep learning (DL) algorithm for classifying three common ED skin lesions and explored whether algorithmic pre-classification was associated with changes in the classification performance of medical staff. **Methods**: A ResNet-50-based algorithm was developed using 6000 clinical images from 4815 patients. For external validation, 100 images from an independent institution were classified by the algorithm and nine medical staff members (three emergency medicine specialists, three residents, and three physician assistant [PA] nurses). Fifteen days later, the staff reclassified the same randomly reordered images with the algorithmic pre-classification provided. **Results**: In external validation, the algorithm showed an overall accuracy of 0.96 (95% confidence interval [CI], 0.90–0.99), lesion-specific balanced accuracies of 0.95–0.96, and a macro-average area under the receiver operating characteristic curve of 0.98 (95% CI, 0.95–1.00). Unassisted, the three PA nurses differed significantly from the algorithm for urticaria and herpes zoster (all *p* < 0.01, McNemar’s exact test). After receiving algorithmic assistance, all medical staff showed a balanced accuracy of 0.97 or higher for all three lesions. In the mixed-effects model, assistance was associated with higher odds of correct classification (odds ratio 25.9, 95% CI 11.7–57.2), and inter-rater agreement (Fleiss’ κ) increased from 0.70 to 0.97. **Conclusions**: The algorithm demonstrated high classification performance on independent external validation, and algorithmic assistance was associated with improved classification performance, with the largest absolute gains among medical staff with lower unassisted accuracy. As these findings are exploratory, they should be confirmed in randomized prospective studies.

## 1. Introduction

Skin conditions are one of the most common reasons for visits to the emergency department (ED) [1,2]. While most skin conditions do not pose an immediate threat to life, the visible nature of the lesions and the discomfort caused by symptoms such as pain or itching can heighten anxiety among patients and their caregivers, creating a strong demand for prompt diagnosis and treatment [2].

During times when dermatology services are limited—such as at night or on holidays—ED medical staff frequently conduct the initial evaluation of patients with skin conditions. In such situations, clinicians with limited dermatology experience may find it difficult to accurately classify lesions, and the accuracy of skin disease recognition by non-dermatologist physicians has been reported to be limited [3]. The diagnosis of skin diseases relies not only on the patient’s clinical symptoms but also on visual characteristics such as the lesion’s shape, color, borders, and distribution; these factors play a crucial role in diagnosis and the decision to consult a specialist [4]. With the widespread adoption of smartphones, teledermatology consultations using clinical images of skin lesions have been utilized as a means to supplement access to specialists [4]. However, this approach can lead to diagnostic delays when an immediate response from a dermatologist is not available, and concerns regarding privacy and information security may arise during the process of capturing and transmitting medical images via personal devices. Therefore, a new approach is needed to support the initial assessment by ED medical staff without relying on real-time intervention by dermatologists.

In recent years, artificial intelligence (AI) and deep learning (DL) have advanced rapidly in the field of medical image analysis and are now being utilized as tools to support the detection and classification of various diseases [5,6].

In the field of dermatology, DL algorithms have demonstrated promising performance in the classification of skin lesions, and some studies have even reported performance comparable to that of board-certified dermatologists for specific diagnostic tasks [7,8,9,10,11]. Esteva et al. trained a convolutional neural network using approximately 130,000 images and reported performance on par with board-certified dermatologists in the binary classification of skin cancer [7]. Fujisawa et al. developed a classifier covering 14 skin tumor diagnoses using fewer than 5000 clinical images collected at a single institution and reported results that outperformed those of board-certified dermatologists [11]. Other studies have extended the application of DL to the differential diagnosis of common skin diseases based on clinical images [10,12,13]. Going beyond the evaluation of standalone algorithms, Jain et al. investigated the use of algorithm-assisted diagnosis when primary care physicians and nurse practitioners reviewed teledermatology cases [14]. A comparison of representative prior studies with the present study is presented in Supplementary Table S1.

Since these studies differ in terms of target diseases, image types, and intended clinical settings, the performance of DL algorithms for evaluating common non-neoplastic skin diseases in the ED cannot be assessed based solely on these findings. In particular, few studies have examined non-dermoscopic clinical images obtained during routine ED care at an independent institution or how algorithm outputs relate to the classification performance of ED medical staff.

Urticaria, herpes zoster, and cellulitis are common skin and soft tissue conditions encountered in EDs in Korea and other countries [1,2,15,16,17]. These conditions are representative examples of allergic-inflammatory, viral, and bacterial diseases, respectively; they require distinct pathophysiological considerations and treatment approaches, and their skin lesions exhibit distinct morphological characteristics. Rapid classification of these conditions in emergency care is crucial for determining appropriate initial treatment. However, for clinicians with limited dermatology experience, accurate classification can be challenging, and misclassification may occur, particularly when there are many clinically similar conditions, such as cellulitis [18,19].

Accordingly, in this study, we developed a multiclass DL algorithm based on non-dermoscopic clinical images of urticaria, herpes zoster, and cellulitis—conditions commonly encountered in the ED—and evaluated its classification performance using an independent external validation dataset. Furthermore, this study compared the classification performance of the algorithm with that of medical staff members with varying levels of clinical experience—including emergency medicine specialists, residents, and physician assistant (PA) nurses—and investigated how the medical staff’s classification performance and inter-rater agreement changed when utilizing the pre-classification information provided by the DL algorithm.

## 2. Materials and Methods

### 2.1. Study Design and Setting

This multicenter, retrospective, preliminary validation study, which evaluated the classification performance of a DL algorithm using non-dermoscopic clinical images of skin lesions collected in emergency departments, was conducted at three emergency medical centers in the Republic of Korea. Data for model development and internal validation were collected from two emergency medical centers, while external validation was performed at an independent third emergency medical center that did not participate in model development.

The study protocol was approved by the Institutional Review Board (IRB) of Yeungnam University Hospital, which served as the central IRB covering all three participating institutions (approval number: YUMC 2024-01-042-001; approved on 26 January 2024). The requirement for individual informed consent was waived due to the retrospective study design. The reporting of this study follows the 2024 update of the Checklist for Artificial Intelligence in Medical Imaging (CLAIM) [20]; the completed checklist is provided in Supplementary Table S2.

### 2.2. Study Population and Dataset Composition

The study population consisted of adult patients aged 18 years or older who visited the EDs of the participating institutions from 1 January 2020 to 31 December 2022 and received a final diagnosis of urticaria, herpes zoster, or cellulitis.

For the model development dataset, 6214 clinical images were identified from 4815 patients at the two emergency medical centers from 1 January 2020 to 31 December 2021. Of these, 214 were excluded according to the exclusion criteria, leaving 6000 clinical images; every one of the 4815 patients contributed at least one included image, and no patient was excluded entirely. The number of images for each disorder was 3450 for urticaria, 1450 for herpes zoster, and 1100 for cellulitis. Of the 6000 images, 5400 were used for training and 600 for internal validation.

**Figure 1:**
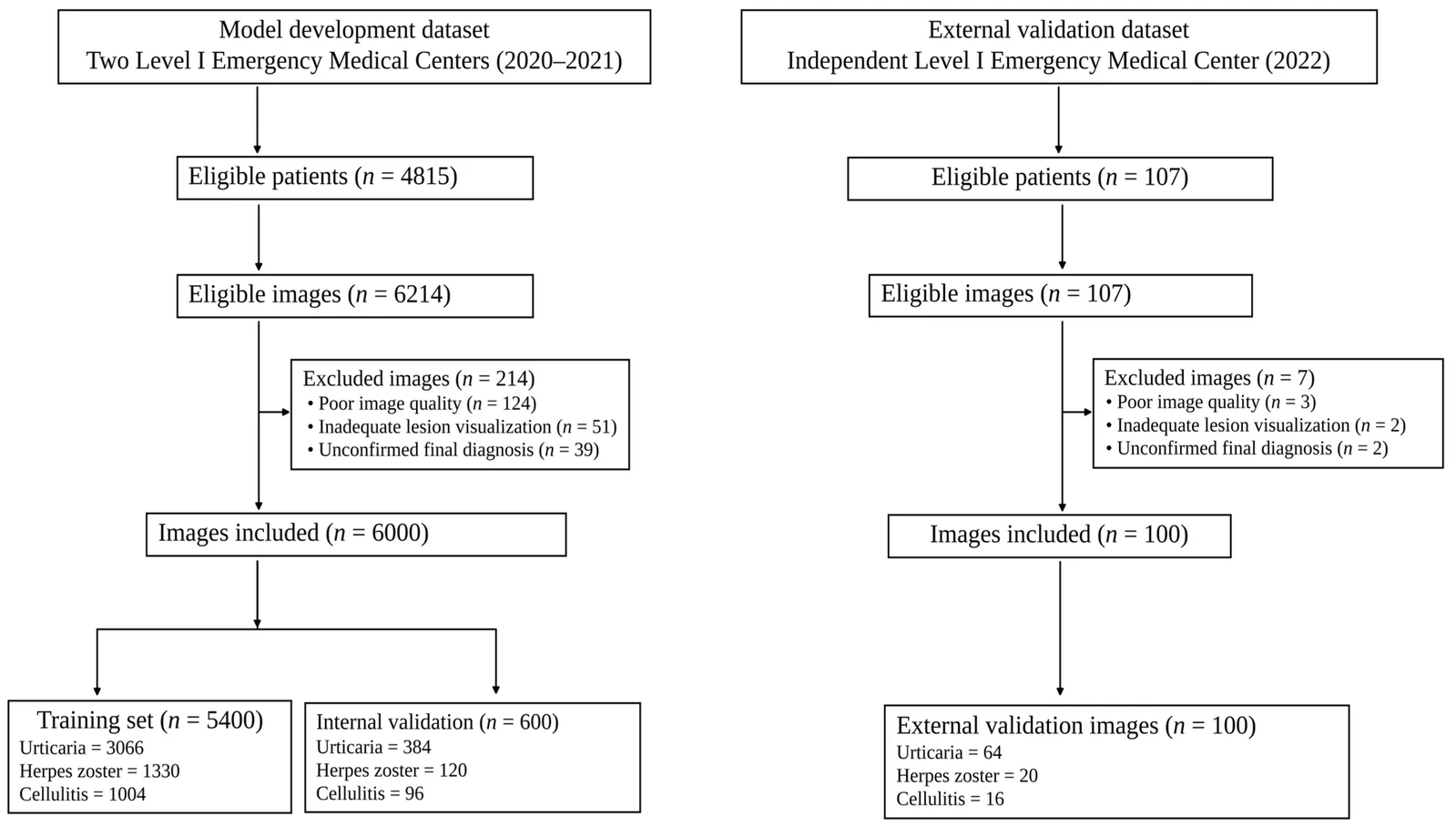
Flow diagram of patient and image selection for model development, internal validation, and external validation. Exclusions were applied at the image level; all 4815 eligible development patients contributed at least one included image.

The external validation dataset comprised 100 clinical images from an independent third institution that had not participated in model development: 64 for urticaria, 20 for herpes zoster, and 16 for cellulitis. These images were obtained after excluding 7 of the 107 images collected from 107 patients between 1 January and 31 December 2022. The screening, exclusion, and final inclusion of the study population are shown in Figure 1.

### 2.3. Image Collection, Preprocessing, and Anonymization

Using the picture archiving and communication system (PACS) and electronic medical records of each participating institution, we identified clinical images and associated clinical information from patients who had received the target diagnoses. Because images were retrieved on the basis of a confirmed target diagnosis rather than by screening consecutive patients presenting with skin complaints, the number of patients with non-target skin diagnoses during the study period was not recorded. Images were taken with the personal smartphone cameras of attending staff under ambient lighting during clinical care; imaging distance and lighting were not standardized, and Fitzpatrick skin type was not recorded.

For the final set of collected images, the investigators confirmed image quality and assessed whether the inclusion and exclusion criteria were met. A quality-control process was performed to select only images in which the lesion was centrally located and clearly in focus. In accordance with predefined exclusion criteria, images in which the lesion could not be visually assessed were excluded from the analysis. The numbers of images excluded for poor image quality, inadequate lesion visualization, and unconfirmed final diagnosis are shown in Figure 1. All collected images were then preprocessed as described in Section 2.4. Information that could directly identify patients was excluded in accordance with the privacy regulations of each institution.

### 2.4. Construction and Internal Validation of the DL Model

The model used the ResNet-50 architecture [21] with ImageNet-pretrained weights, with the final fully connected layer replaced for three-class classification of urticaria, herpes zoster, and cellulitis. Images were obtained by capturing a 224 × 224-pixel square region containing the lesion from the PACS display, on which each image was shown at its original aspect ratio; no resizing or padding was applied. Captures were saved in RGB format, and the same procedure was applied across the training, internal validation, and external validation datasets. Data augmentation, class weighting, and resampling were not applied.

The 6000 development images were randomly partitioned at the patient level into 5400 training images and 600 internal validation images, without class stratification. No patient contributed images to both sets. The model was trained for up to 100 epochs using a batch size of 16, the Adam optimizer, categorical cross-entropy loss, and an initial learning rate of 0.0001. Validation loss guided early stopping, learning-rate adjustment, and selection of the final checkpoint; the checkpoint with the lowest internal validation loss was selected (internal validation accuracy 0.98, 586/600), a development-stage estimate rather than independent test performance.

The model was implemented using TensorFlow 2.17 (Google LLC, Mountain View, CA, USA) in Google Colaboratory (https://colab.research.google.com), with the random seed set to 42. The external validation set was not used for training, early stopping, learning-rate adjustment, checkpoint selection, or hyperparameter optimization. The same fixed final model was used for external validation and to generate the pre-classification labels presented to the medical staff during the assisted assessment.

The reference standard was the treating dermatologist’s final diagnosis documented during routine care before model development, based on the full clinical context rather than the image alone. When multiple assessments were available, the diagnosis at follow-up, discharge, or the final visit was used. Cellulitis was diagnosed based on clinical findings, disease course, and response to antibiotic treatment. The dermatologists were unaware of the algorithm’s output. No independent re-adjudication was performed, and patients without a dermatologist-confirmed diagnosis were excluded.

### 2.5. External Validation and Assessment of Medical Staff Classification Performance

The DL algorithm and medical staff classified each of the 100 external validation images as urticaria, herpes zoster, or cellulitis using images alone. The algorithm assigned the class with the highest softmax probability. Class probabilities were obtained by repeating inference using the saved final model and identical preprocessing, without retraining, fine-tuning, or recalibration. The resulting classifications were compared with the original predictions used in the medical staff assessment.

Nine medical staff members (three emergency medicine specialists with 13, 6, and 3 years of post-certification experience; three residents in their first, second, and third years of training; and three PA nurses with 6, 5, and 2 years of ED experience) were recruited by convenience sampling from the EDs of the participating institutions; none had ever worked at the external validation institution. The PA nurses were licensed nurses involved in initial patient assessment, assistance with medical care and procedures, and clinical monitoring under physician supervision.

Each medical staff member independently classified the images in a DL algorithm–unassisted phase and, after a 15-day washout period, in a DL algorithm–assisted phase in which the algorithm’s classification result was displayed to the right of each image without probability information. Image order was randomized independently for each participant; participants were not required to follow the algorithm, were blinded to the reference standard, and received no feedback after the unassisted phase.

Gradient-weighted class activation mapping (Grad-CAM) [22] maps for the predicted class were generated from the final convolutional block (conv5_block3) of the fixed model, resized to the input resolution, and overlaid on the original images.

### 2.6. Outcome Measures

The primary outcome was the performance of the DL algorithm in classifying urticaria, herpes zoster, and cellulitis using an independent external validation dataset. Overall multiclass performance was evaluated using accuracy, Cohen’s κ, the macro F1 score, and the multiclass Matthews correlation coefficient (MCC). Lesion-specific performance was evaluated using a one-versus-rest approach, with sensitivity, specificity, positive predictive value (PPV), negative predictive value (NPV), F1 score, MCC, and balanced accuracy calculated for each disorder. Balanced accuracy was defined as the mean of sensitivity and specificity. Discrimination was assessed using class-specific one-versus-rest areas under the receiver operating characteristic (ROC) curve (AUCs) and the macro-average AUC, defined as the unweighted mean of the three class-specific AUCs. Calibration was assessed using a reliability diagram and the expected calibration error (ECE), and overall probabilistic performance was summarized using the multiclass Brier score. An exploratory confidence-threshold analysis, in which images with a maximum predicted probability below a threshold are deferred, assessed coverage, accuracy among retained images, and the number of algorithm misclassifications deferred.

The secondary outcomes were the classification performance of each medical staff member compared with that of the DL algorithm in the DL algorithm–unassisted and DL algorithm–assisted phases, the within-staff change in correct or incorrect classification, and the change in agreement among medical staff before and after DL algorithm assistance.

### 2.7. Statistical Analysis

Continuous variables were summarized as medians with interquartile ranges (IQRs) and categorical variables as frequencies and percentages. Datasets were compared using the Mann–Whitney U test for age and the chi-square or Fisher’s exact test for categorical variables.

For external validation, 95% confidence intervals (CIs) for accuracy, sensitivity, specificity, PPV, and NPV were calculated using the Clopper–Pearson exact binomial method. CIs for balanced accuracy, Cohen’s κ, and Fleiss’ κ were obtained from 5000 image-level bootstrap resamples using the percentile method in Python (NumPy 2.0.1). Each external validation image represented a different patient. F1 scores and MCCs were reported as point estimates.

Recorded class probabilities were converted from integer percentages to proportions. Class-specific and macro-average AUC CIs were calculated using the percentile method from 5000 patient-level bootstrap resamples stratified by reference-standard class (Python, NumPy and scikit-learn). For calibration analysis, three confidence groups (0.50–0.79, 0.80–0.99, and 1.00) were defined after inspection of the maximum predicted probability distribution. Reliability diagrams plotted mean predicted probability against observed accuracy, with Wilson 95% CIs for accuracy. ECE was calculated as the sample-size-weighted mean absolute difference between these quantities. The multiclass Brier score was calculated as the mean sum of squared differences between predicted probabilities and one-hot-encoded reference labels (range, 0–2). Exploratory confidence thresholds from 0.50 to 0.99 were evaluated using the measures defined in Section 2.6.

Lesion-specific comparisons between the algorithm and each medical staff member used McNemar’s exact test on paired one-versus-rest classifications. These tests assessed discordant classifications rather than differences in balanced accuracy.

The primary analysis of within-staff change used mixed-effects logistic regression on 1800 readings (100 images × 9 medical staff members × 2 phases), with correct classification as the outcome, assistance and staff role as fixed effects, and crossed random intercepts for medical staff and image. Models were fitted by maximum likelihood with Laplace approximation. The assistance-by-role interaction was assessed using a likelihood-ratio test and omitted if not significant. Sensitivity analyses examined alternative model specifications and participant or image subsets, a parametric bootstrap CI (500 replicates), and a generalized estimating equation (GEE) with exchangeable correlation within images (Supplementary Table S3). Secondary within-staff comparisons used McNemar’s exact test with Holm adjustment across the nine medical staff members.

No prior sample size calculation was performed, and inferential analyses were considered exploratory. Lesion-specific algorithm–staff comparisons were not adjusted for multiplicity and were interpreted with recognition of the increased risk of Type I error. All tests were two-sided, with a significance threshold of 0.05. Analyses used MedCalc 19.4.1 (MedCalc Software Ltd, Ostend, Belgium), IBM SPSS Statistics 26.0 (IBM Corp., Armonk, NY, USA), Python 3.11 (https://www.python.org/) with NumPy 2.0.1 and scikit-learn 1.5.2 (random seed 42), and R 4.3.3 (R Foundation for Statistical Computing, Vienna, Austria) with lme4 1.1-35.

## 3. Results

### 3.1. Study Population and Dataset Characteristics

The model development dataset comprised 6000 skin lesion images obtained from 4815 patients at two regional emergency medical centers. The independent external validation dataset comprised 100 skin lesion images from 100 patients collected at a third emergency medical institution that had not participated in model development (Figure 1).

The median age was 52 years (IQR, 36–66) in the model development group and 48.5 years (IQR, 36–59) in the external validation group, with no significant difference in age distribution between the two groups (*p* = 0.67). The proportions of men were 42.2% and 36.0%, respectively, with no significant difference in sex distribution (*p* = 0.21). The demographic characteristics and disorder distributions of the two datasets are presented in Table 1.

### 3.2. External Validation Performance of the DL Algorithm

The external validation performance of the DL algorithm is presented in Table 2. The fixed final model correctly classified 96 of the 100 images (overall accuracy 0.96; 95% CI, 0.90–0.99). Lesions were located on the trunk in 44, the lower limb in 26, the upper limb in 20, and the head and neck in 10 images.

Lesion-specific balanced accuracies were 0.96, 0.95, and 0.96 for urticaria, herpes zoster, and cellulitis (macro-average 0.96); F1 scores were 0.98, 0.95, and 0.91 (macro F1 0.94); and MCCs were 0.93, 0.94, and 0.89 (multiclass MCC 0.92).

**Figure 2:**
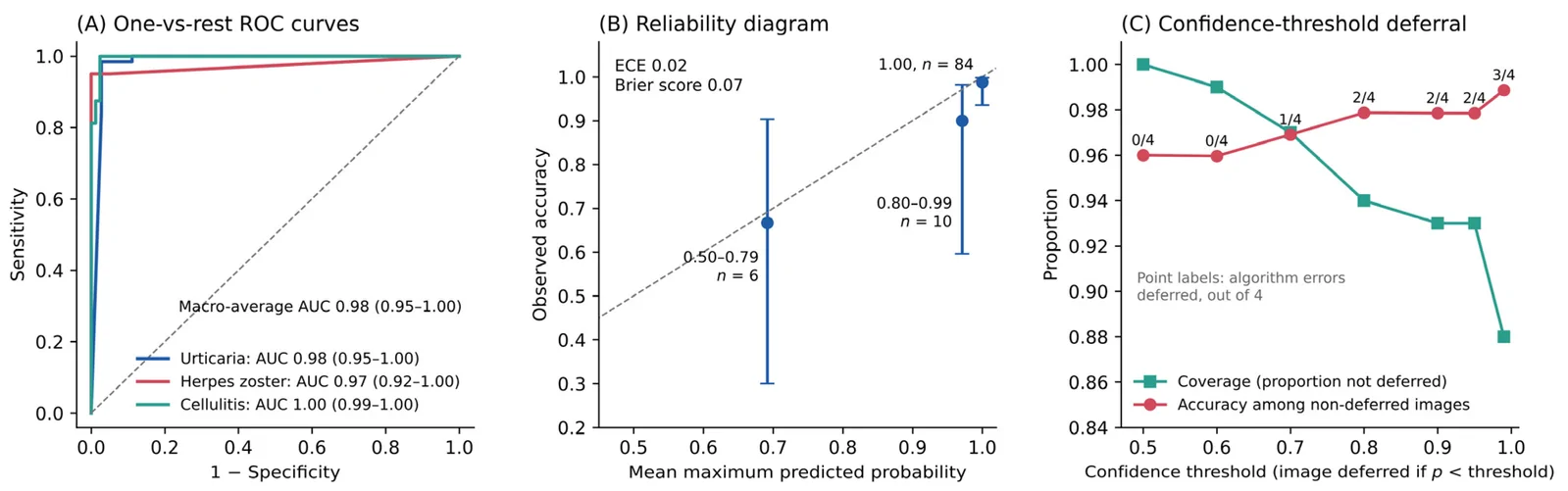
Discrimination, calibration, and confidence-threshold analysis of the fixed final model in the external validation set (*n* = 100). (**A**) One-vs.-rest receiver operating characteristic (ROC) curves for urticaria, herpes zoster, and cellulitis. (**B**) Reliability diagram of the maximum predicted probability. (**C**) Exploratory confidence-threshold analysis showing coverage and accuracy among retained images when deferring predictions below specified thresholds. The gray dashed diagonal line indicates chance-level discrimination in panel (**A**) and perfect calibration in panel (**B**). Error bars in panel (**B**) indicate Wilson 95% confidence intervals. AUC, area under the curve; ECE, expected calibration error.

Repeated inference reproduced the original algorithm classifications for all 100 images. The one-versus-rest AUCs were 0.98 (95% CI, 0.95–1.00) for urticaria, 0.97 (95% CI, 0.92–1.00) for herpes zoster, and 1.00 (95% CI, 0.99–1.00; below 1.00 before rounding) for cellulitis, with a macro-average AUC of 0.98 (95% CI, 0.95–1.00) (Figure 2A).

The recorded maximum predicted probability was 1.00 for 84 images; observed accuracy was 0.67 (95% CI, 0.30–0.90; *n* = 6) in the 0.50–0.79 group, 0.90 (95% CI, 0.60–0.98; *n* = 10) in the 0.80–0.99 group, and 0.99 (95% CI, 0.94–1.00; *n* = 84) in the 1.00 group (Figure 2B); the ECE was 0.02 and the Brier score 0.07. The four misclassified images had maximum probabilities of 0.69, 0.73, 0.97, and 1.00. At a threshold of 0.80, 6 images were deferred (coverage 94%), accuracy among retained images was 0.98 (92/94), and two of the four misclassifications were deferred; at 0.99, 12 were deferred and retained accuracy was 0.99 (87/88). The herpes zoster image misclassified with a probability of 1.00 was not deferred at any evaluated threshold (Figure 2C).

**Figure 3:**
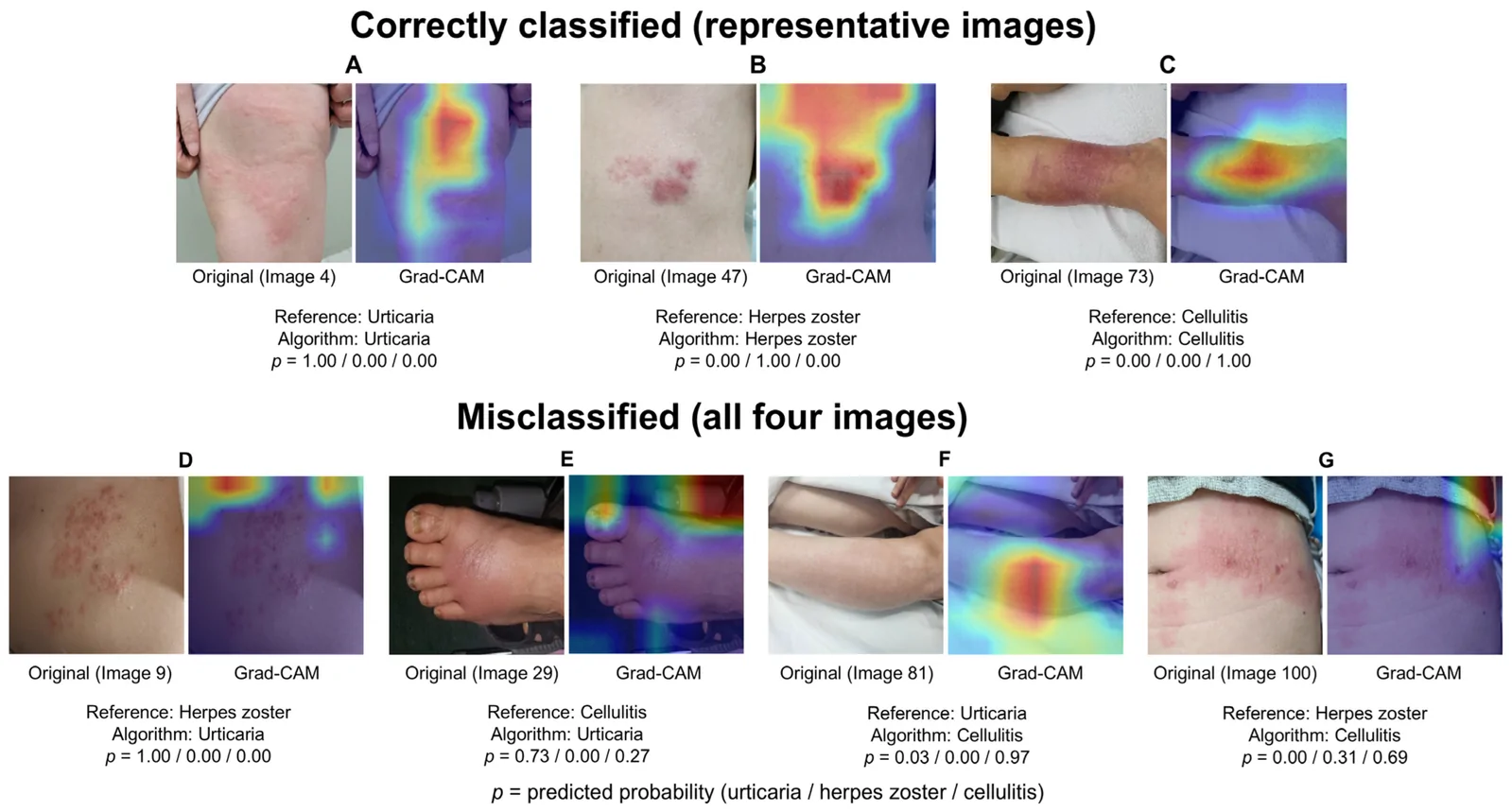
Gradient-weighted class activation maps (Grad-CAM) of the fixed final model in the external validation set. For each case, the original image (left) and the Grad-CAM heat map (right) are shown alongside the reference diagnosis, the algorithm’s classification, and the predicted probabilities for urticaria, herpes zoster, and cellulitis. Warmer colors indicate higher activation. (**A**–**C**) Representative correctly classified images. (**D**–**G**) All four misclassified images (Images 9, 29, 81, and 100).

Grad-CAM maps are shown in Figure 3. In the representative correctly classified images, heat-map activation was centered on the lesion. In three of the four misclassified images, activation lay predominantly outside the lesion—on background skin (Image 9, herpes zoster classified as urticaria, 1.00), a toenail and image edge (Image 29, cellulitis classified as urticaria, 0.73), and adjacent clothing (Image 100, herpes zoster classified as cellulitis, 0.69)—whereas in Image 81 (urticaria classified as cellulitis, 0.97) activation was localized to the skin lesion, an ill-defined erythematous patch.

### 3.3. External Validation Performance of the Medical Staff

The lesion-specific classification performance of the medical staff in the external validation dataset is presented in Table 2.

For urticaria, sensitivity was 0.94–0.98 for the specialists, 0.86–0.97 for the residents, and 0.61–0.62 for the PA nurses, whereas specificity was 1.00, 0.97, and 0.94–1.00, respectively. PPV was 1.00 for all specialists, 0.98 for all residents, and 0.95–1.00 for the PA nurses.

For herpes zoster, the sensitivity and specificity of the specialists were 1.00 and 0.95–1.00, respectively. Among the residents, sensitivity was 1.00 for all, and specificity was 0.90–0.99. The sensitivity of the PA nurses was high at 0.90–0.95, whereas specificity was 0.81–0.84 and PPV was 0.55–0.59.

For cellulitis, sensitivity was 0.94–1.00 for the specialists and 0.94 for all residents, and specificity was 0.99 for all specialists and 0.98–0.99 for the residents. Among the PA nurses, sensitivity was 1.00 for all three staff members, whereas specificity was 0.87–0.88 and PPV was 0.59–0.62.

Among the PA nurses, sensitivity for urticaria (0.61–0.62) was lower than that for herpes zoster (0.90–0.95) and cellulitis (1.00). Conversely, despite the high sensitivity for herpes zoster and cellulitis, specificity and PPV were relatively low.

### 3.4. Agreement of the DL Algorithm and Medical Staff with the Reference Standard

Agreement (Cohen’s κ) between the reference standard and the classifications of the algorithm and medical staff is presented in Table 3. The algorithm showed κ = 0.92. Agreement with the reference standard was highest for the specialists (κ = 0.91–0.98) and the third-year resident (κ = 0.94), whereas it was lower for the first- and second-year residents (κ = 0.82–0.85) and the PA nurses (κ = 0.57–0.60). Agreement among the nine medical staff (Fleiss’ κ) was 0.70.

### 3.5. Medical Staff Performance Before DL Algorithm Assistance

The overall accuracy and lesion-specific balanced accuracy of the medical staff in the DL algorithm–unassisted phase are presented in Table 4. Overall accuracy was 0.95–0.99 for the specialists, 0.90–0.97 for the residents, and 0.73–0.75 for the PA nurses.

For urticaria, balanced accuracy was 0.97–0.99 for the specialists, 0.92–0.97 for the residents, and 0.78–0.81 for the PA nurses, compared with 0.96 for the DL algorithm. For herpes zoster, the corresponding values were 0.97–1.00, 0.95–0.99, and 0.86–0.89, respectively, compared with 0.95 for the algorithm. For cellulitis, balanced accuracy was 0.96–0.99 for the specialists, 0.96 for the residents, and 0.94 for the PA nurses, compared with 0.96 for the algorithm.

In paired image-level comparisons, the classifications of all three PA nurses differed significantly from those of the DL algorithm for urticaria and herpes zoster (all *p* < 0.01), whereas no significant differences were detected between the algorithm and any specialist or resident. For cellulitis, no significant difference was detected for any medical staff member (*p* = 0.06–1.00).

### 3.6. Medical Staff Performance After DL Algorithm Assistance

In the DL algorithm–assisted phase, overall accuracy was 0.99–1.00 for the specialists, 0.98–0.99 for the residents, and 0.97 for the PA nurses (Table 5). Lesion-specific balanced accuracy was 0.97 or higher for all medical staff across the three disorders. In the paired image-level comparisons, no significant differences from the DL algorithm were detected for any medical staff member for urticaria, herpes zoster, or cellulitis (all *p* ≥ 0.25).

### 3.7. Comparison of Medical Staff Performance Before and After DL Algorithm Assistance

In the mixed-effects model, the assisted phase was associated with higher odds of correct classification (odds ratio [OR] 25.9, 95% CI 11.7–57.2; *p* < 0.001), with no significant assistance-by-role interaction (*p* = 0.60). Compared with specialists, the odds of correct classification were lower for residents (OR 0.22, 95% CI 0.08–0.57) and PA nurses (OR 0.03, 95% CI 0.01–0.07). The medical-staff random-effect variance was estimated at zero. Sensitivity analyses showed associations in the same direction, with ORs of 23.5–26.3 in the mixed-effects models and 10.6 in the GEE analysis (Supplementary Table S3).

**Figure 4:**
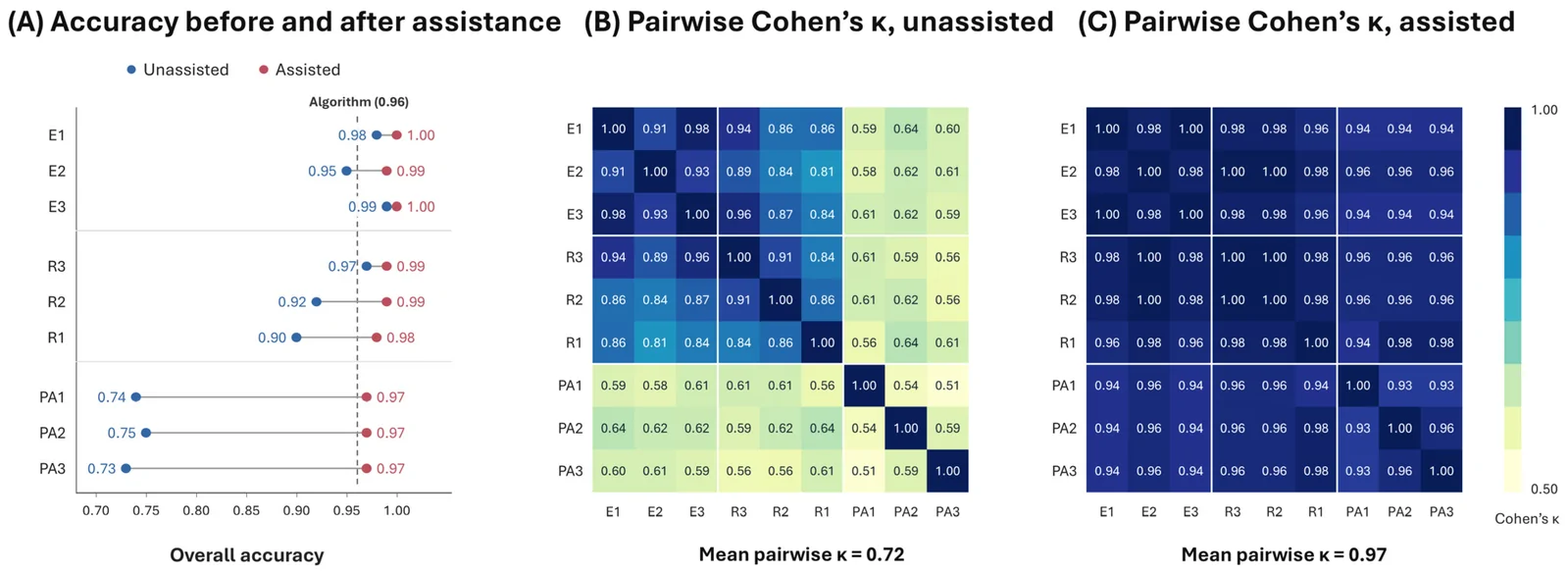
Medical staff performance in skin lesion classification and inter-staff agreement before and after DL algorithm assistance. (**A**) Overall accuracy of skin lesion classification by each medical staff member in the DL algorithm–unassisted (blue) and DL algorithm–assisted (red) phases; the dashed vertical line indicates the standalone classification accuracy of the DL algorithm (0.96). (**B**,**C**) Pairwise Cohen’s κ among the nine medical staff members in the DL algorithm–unassisted (**B**) and DL algorithm–assisted (**C**) phases. DL, deep learning; E1–E3, emergency medicine specialists; R1–R3, emergency medicine residents (first to third year); PA1–PA3, physician assistant nurses.

Absolute increases in accuracy were 0.01–0.04 for specialists, 0.02–0.08 for residents, and 0.22–0.24 for PA nurses. Accuracy across medical staff ranged from 0.73 to 0.99 before assistance and from 0.97 to 1.00 during assistance (Figure 4A). Fleiss’ κ increased from 0.70 (95% CI, 0.62–0.77) to 0.97 (95% CI, 0.94–0.99), and mean pairwise Cohen’s κ increased from 0.72 to 0.97 (Figure 4B,C).

**Figure 5:**
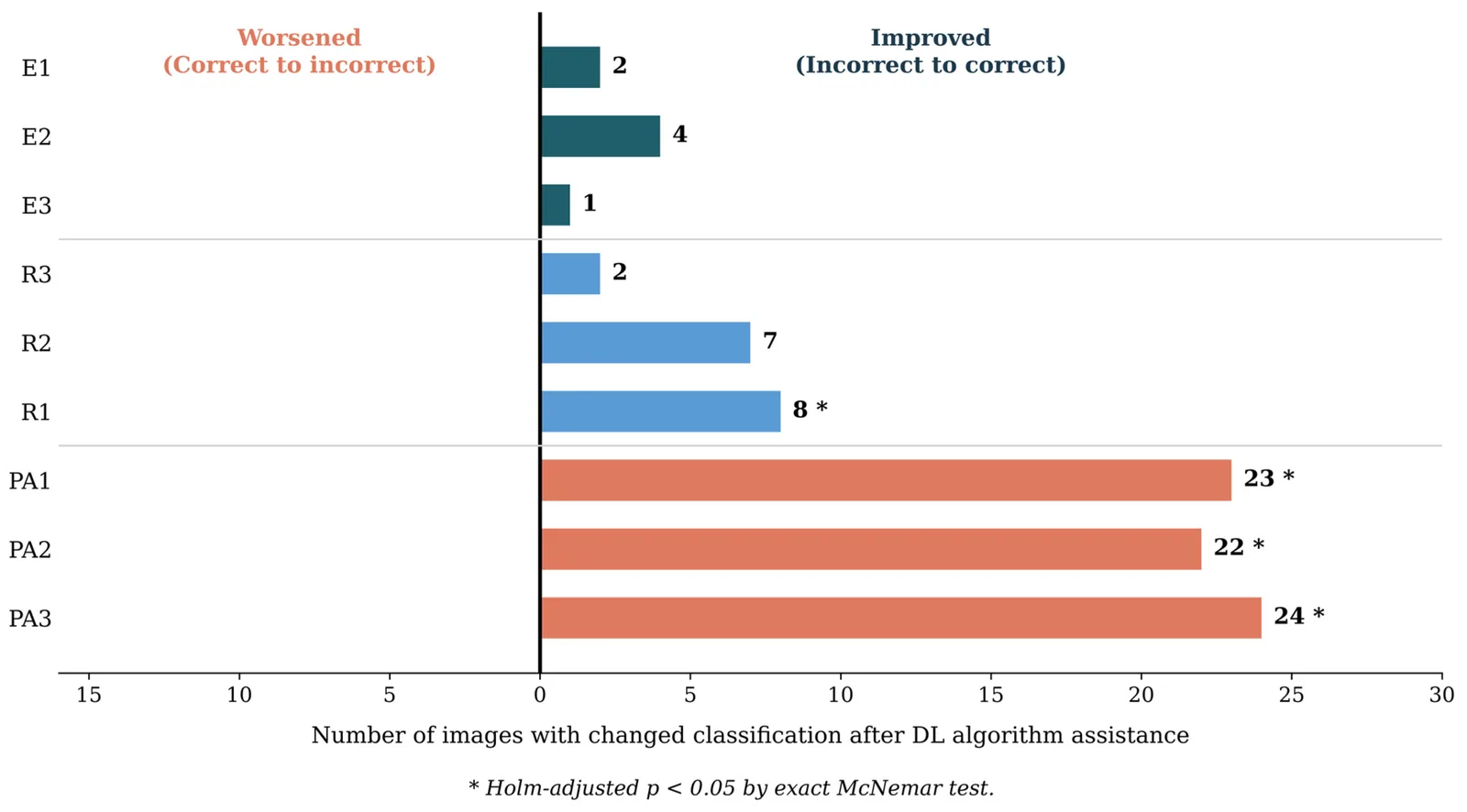
Image-level classification changes for each medical staff member after DL algorithm assistance: the number of the 100 external validation images that changed from incorrect to correct or from correct to incorrect between the DL algorithm–unassisted and DL algorithm–assisted phases. Asterisks indicate Holm-adjusted *p* < 0.05 by McNemar’s exact test. Bar colors indicate staff roles: dark teal for emergency medicine specialists, blue for emergency medicine residents, and orange for physician assistant nurses. DL, deep learning; E1–E3, emergency medicine specialists; R1–R3, emergency medicine residents (first to third year); PA1–PA3, physician assistant nurses.

All 93 classification changes were from incorrect to correct; 92 matched the algorithm’s label. After Holm adjustment, changes were significant for the first-year resident (*p* = 0.047) and all three PA nurses (all *p* < 0.001), but not for the second-year resident (*p* = 0.08), third-year resident, or specialists (Figure 5). The one change that did not match the algorithm’s label occurred in Image 81, which the algorithm misclassified as cellulitis: PA3 classified this image as cellulitis without assistance and correctly as urticaria with assistance, despite the algorithm’s incorrect label. All other classifications of the four algorithm-misclassified images were correct in both phases.

## 4. Discussion

In this multicenter, retrospective, preliminary validation study, an image-based ResNet-50 DL algorithm classified urticaria, herpes zoster, and cellulitis from non-dermoscopic clinical images with an overall accuracy of 0.96 (95% CI, 0.90–0.99) and a macro-average AUC of 0.98 in an independent external validation dataset. The algorithm-assisted phase was associated with higher odds of correct classification and greater agreement among medical staff. The largest absolute increases in accuracy were observed among PA nurses, whose unassisted performance was lowest. These findings suggest a potential role for algorithmic pre-classification in supporting image-based assessment of selected skin lesions in the ED.

An important feature of this study was the use of clinical images obtained during routine ED care, with external validation at an institution that did not contribute to model development. Previous dermatology AI studies have addressed skin tumors and the broader differential diagnosis of skin conditions using dermoscopic or clinical images, including assessments in dermatology and primary care settings [7,8,9,10,11,14] (Supplementary Table S1). Clinical images obtained in the ED vary in lighting, distance, angle, background, and resolution, creating challenges for image-based classification [8]. The present study examined three non-neoplastic disorders commonly encountered in emergency care and evaluated both algorithm performance and medical staff classifications with and without algorithm assistance. However, the selected images and small external validation sample provide only preliminary evidence of performance in this setting [23].

Medical staff with lower unassisted accuracy showed larger absolute improvements during the assisted phase. After Holm adjustment, changes remained significant for the first-year resident and all three PA nurses, whereas the change for the second-year resident was not significant. Specialists and the third-year resident had high unassisted accuracy, leaving relatively little room for improvement. Nevertheless, the assistance-by-role interaction was not significant, and these findings do not establish that the association between assistance and correct classification differed across staff roles. The observed pattern supports further evaluation of algorithmic assistance among medical staff with varying baseline performance.

The lesion-specific findings provide additional clinical context. Before assistance, PA nurses had lower sensitivity for urticaria (0.61–0.62) than for herpes zoster (0.90–0.95) or cellulitis (1.00). Although typical wheals may be readily recognized, urticaria varies in morphology and distribution, changes over time, and has several clinical mimics [24]. Assessment from a single image excludes information such as pruritus, symptom duration, and lesion evolution that may help distinguish these conditions. The improvement in PA nurses’ classification of urticaria is therefore an exploratory finding of clinical interest. Previous studies have examined DL-based and algorithm-assisted classification of skin disorders, including in primary care settings [14,25]; the present study extends this evaluation to selected acute skin lesions encountered in the ED. However, the specific morphological features underlying the model’s predictions were not systematically evaluated, and the findings do not establish that the model recognizes urticaria more accurately or objectively than clinicians.

High image-based classification performance should not be equated with diagnostic performance in clinical practice. The reference diagnoses were established by dermatologists using the clinical context in addition to lesion appearance, whereas both the algorithm and medical staff were evaluated using images alone. Symptom onset and course, pain or pruritus, fever, lesion location, examination findings, medication history, and underlying conditions may all contribute to diagnosis. Multimodal approaches combining clinical images with structured clinical information may better reflect this process, and improved performance over image-only approaches has been reported in other skin disorder classification tasks [26]. The incremental value of clinical information was not evaluated here and should be examined through direct comparisons of image-only and multimodal models.

Agreement among medical staff increased during the assisted phase, with Fleiss’ κ rising from 0.70 to 0.97 and mean pairwise Cohen’s κ from 0.72 to 0.97. More consistent classifications may be useful in an ED where staff have differing levels of experience. However, agreement alone does not establish diagnostic validity, because a shared algorithmic error could influence multiple staff members in the same direction. Most answer changes (92/93) were toward the algorithm’s label, but this observation does not establish either susceptibility to or resistance to automation bias. With one exception (Image 81, which one PA nurse misclassified without assistance and then corrected despite the algorithm’s incorrect label), every medical staff member had already classified the four algorithm-misclassified images correctly without assistance. Consequently, the absence of correct-to-incorrect changes provides little information about responses to plausible algorithm errors on images that staff find difficult.

The error analysis also identified concerns relevant to clinical application. Grad-CAM highlighted non-lesional regions in three of the four misclassified images, suggesting that background cues may have contributed to these predictions. These maps do not establish the cause of misclassification or exclude confusion between lesion appearances. One misclassified image had a recorded maximum predicted probability of 1.00, illustrating that a high model confidence score does not guarantee a correct classification. Future studies could examine whether lesion-focused preprocessing or augmentation that preserves diagnostically relevant features reduces sensitivity to non-lesional image content.

The three-class design further limits the scope of the algorithm. Images of contact dermatitis, erysipelas, drug eruption, deep vein thrombosis, and other mimics would still be assigned to one of the three trained categories. In the exploratory threshold analysis, a threshold of 0.80 retained 94% of images and deferred two of the four misclassified images. However, the error with a recorded probability of 1.00 was not deferred at any evaluated threshold. Moreover, because no mimics were included in the external validation set, the analysis cannot establish whether confidence thresholds would identify non-target disorders. Training and validation with a broader diagnostic spectrum, potentially including an “other” category and an uncertainty-based deferral strategy, are needed before considering broader clinical use. The present algorithm should therefore not be used as a stand-alone triage tool.

Several limitations should be considered. First, the external validation set contained only 100 images from a single institution, including 20 images of herpes zoster and 16 of cellulitis, limiting the precision of lesion-specific estimates. All patients were Korean. Fitzpatrick skin type was unavailable for both datasets, and lesion location was unavailable for the development dataset. Generalizability across populations, skin types, and imaging conditions remains unknown. Diagnosis-based retrieval and exclusion of inadequate-quality or diagnostically uncertain cases also introduced spectrum and selection bias. Neither the total number of ED skin-lesion presentations nor the number of patients with non-target diagnoses was available. In addition, investigator selection of the 224 × 224-pixel capture region may have excluded surrounding clinical information. Performance in an unselected ED population may therefore differ from that observed here.

Second, the sequential assessment of the same images could not separate algorithm assistance from recall or learning effects, despite the 15-day washout period, independently randomized image order, and absence of feedback after the unassisted phase. A counterbalanced design or an unassisted repeat-assessment control is needed to distinguish these effects. The convenience sample of nine medical staff members, with three per role, also limits generalizability and the precision of between-role comparisons. The medical-staff random-effect variance was estimated at zero; although sensitivity analyses showed associations in the same direction, the small number of participants remains a limitation. Nonsignificant role differences should not be interpreted as evidence of equivalence. Holm adjustment was applied to the nine within-staff comparisons, but the unadjusted lesion-specific algorithm–staff comparisons remain susceptible to Type I error from multiple testing.

Third, model development used a single, non-stratified patient-level partition, with 8.3–11.1% of the images from each disorder allocated to the internal validation set. The internal validation set guided early stopping and checkpoint selection and therefore did not provide an independent estimate of performance. External validation of the fixed model does not replace assessment of variability across development-data partitions, which was not examined using cross-validation. Data augmentation, class weighting, and resampling were not applied, as the study aimed to establish the baseline performance of a standard transfer-learning pipeline. The training set was relatively large, and validation loss converged steadily. However, this convergence does not establish robustness, and the effects of these choices on overall or minority-class performance were not evaluated. The absence of augmentation may have reduced robustness to variation in imaging conditions, although the Grad-CAM findings cannot be attributed to it. ResNet-50 was selected for its availability and ease of implementation, but lighter architectures such as ResNet-18, MobileNetV2, or EfficientNet-B0 were not compared. Hardware specifications and training time were not recorded, preventing assessment of computational cost.

Fourth, the probability-based analyses used values recorded as integer percentages, limiting precision and introducing ties. Calibration groups were defined after inspection of the probability distribution, and the two lower-confidence groups contained few images. The reliability diagram and ECE should therefore be interpreted cautiously, and the low ECE does not establish reliable calibration in other samples. Confidence thresholds were explored in the same external validation set and were not validated independently.

Finally, the reference standard was the treating dermatologist’s documented diagnosis, without independent re-adjudication or assessment of inter-adjudicator agreement. The study evaluated image-based classification rather than comprehensive diagnosis and did not measure medical staff reading time or effects on treatment decisions, dermatology consultation, or patient outcomes. Larger prospective studies incorporating clinical information and a broader range of presentations are needed to determine whether the observed classification changes translate into clinical benefit. A confirmatory study is planned that will include patient-level cross-validation, comparison of models trained with and without augmentation, an “other” category with common mimics, and a design that deliberately includes plausible algorithm errors.

## 5. Conclusions

In summary, the image-based DL algorithm showed high classification performance for urticaria, herpes zoster, and cellulitis in an independent external validation dataset, with an overall accuracy of 0.96 (95% CI, 0.90–0.99) and a macro-average AUC of 0.98. The subsequent algorithm-assisted phase was associated with higher odds of correct classification (odds ratio, 25.9; 95% CI, 11.7–57.2), with the largest absolute gains among PA nurses and junior residents, and the mean pairwise Cohen’s κ among medical staff increased from 0.72 to 0.97. These findings suggest the potential value of DL algorithm–based pre-classification as a supportive tool for medical staff with lower unassisted classification accuracy. However, the algorithm cannot be used as a stand-alone triage tool. These exploratory findings require confirmation in larger multicenter prospective studies.

## Figures and Tables

**Table 1:** Demographic characteristics and dataset distribution.

Variables	Model Development Cohort (*n* = 4815 Patients)	External Validation Cohort (*n* = 100 Patients)	*p*
Age, years, median (IQR)	52 (36–66)	48.5 (36–59)	0.67
Sex, *n* (%)			0.21
Male	2034 (42.2)	36 (36.0)	
Female	2781 (57.8)	64 (64.0)	
Final diagnosis, *n* (%)			0.91
Urticaria	2979 (61.9)	64 (64.0)	
Herpes zoster	1027 (21.3)	20 (20.0)	
Cellulitis	809 (16.8)	16 (16.0)	

IQR, interquartile range.

**Table 2:** Lesion-specific classification performance of the DL algorithm and nine medical staff members in the external validation dataset.

Lesion	Medical Staff	Sensitivity	Specificity	PPV	NPV	F1	MCC
Urticaria	DL algorithm	0.98 (0.92–1.00)	0.94 (0.81–0.99)	0.97 (0.89–1.00)	0.97 (0.85–1.00)	0.98	0.93
	E1	0.97 (0.89–1.00)	1.00 (0.90–1.00)	1.00 (0.94–1.00)	0.95 (0.82–0.99)	0.98	0.96
	E2	0.94 (0.85–0.98)	1.00 (0.90–1.00)	1.00 (0.94–1.00)	0.90 (0.76–0.97)	0.97	0.92
	E3	0.98 (0.92–1.00)	1.00 (0.90–1.00)	1.00 (0.94–1.00)	0.97 (0.86–1.00)	0.99	0.98
	R3	0.97 (0.89–1.00)	0.97 (0.85–1.00)	0.98 (0.91–1.00)	0.95 (0.82–0.99)	0.98	0.94
	R2	0.89 (0.79–0.95)	0.97 (0.85–1.00)	0.98 (0.91–1.00)	0.83 (0.69–0.93)	0.93	0.84
	R1	0.86 (0.75–0.93)	0.97 (0.85–1.00)	0.98 (0.90–1.00)	0.80 (0.65–0.90)	0.92	0.80
	PA1	0.61 (0.48–0.73)	1.00 (0.90–1.00)	1.00 (0.91–1.00)	0.59 (0.46–0.71)	0.76	0.60
	PA2	0.62 (0.50–0.74)	0.97 (0.85–1.00)	0.98 (0.87–1.00)	0.59 (0.46–0.72)	0.76	0.58
	PA3	0.61 (0.48–0.73)	0.94 (0.81–0.99)	0.95 (0.83–0.99)	0.58 (0.44–0.70)	0.74	0.54
Herpes zoster	DL algorithm	0.90 (0.68–0.99)	1.00 (0.95–1.00)	1.00 (0.81–1.00)	0.98 (0.91–1.00)	0.95	0.94
	E1	1.00 (0.83–1.00)	0.99 (0.93–1.00)	0.95 (0.76–1.00)	1.00 (0.95–1.00)	0.98	0.97
	E2	1.00 (0.83–1.00)	0.95 (0.88–0.99)	0.83 (0.63–0.95)	1.00 (0.95–1.00)	0.91	0.89
	E3	1.00 (0.83–1.00)	1.00 (0.95–1.00)	1.00 (0.83–1.00)	1.00 (0.95–1.00)	1.00	1.00
	R3	1.00 (0.83–1.00)	0.99 (0.93–1.00)	0.95 (0.76–1.00)	1.00 (0.95–1.00)	0.98	0.97
	R2	1.00 (0.83–1.00)	0.94 (0.86–0.98)	0.80 (0.59–0.93)	1.00 (0.95–1.00)	0.89	0.87
	R1	1.00 (0.83–1.00)	0.90 (0.81–0.96)	0.71 (0.51–0.87)	1.00 (0.95–1.00)	0.83	0.80
	PA1	0.95 (0.75–1.00)	0.81 (0.71–0.89)	0.56 (0.38–0.73)	0.98 (0.92–1.00)	0.70	0.64
	PA2	0.95 (0.75–1.00)	0.84 (0.74–0.91)	0.59 (0.41–0.76)	0.99 (0.92–1.00)	0.73	0.68
	PA3	0.90 (0.68–0.99)	0.81 (0.71–0.89)	0.55 (0.36–0.72)	0.97 (0.90–1.00)	0.68	0.61
Cellulitis	DL algorithm	0.94 (0.70–1.00)	0.98 (0.92–1.00)	0.88 (0.64–0.99)	0.99 (0.93–1.00)	0.91	0.89
	E1	1.00 (0.79–1.00)	0.99 (0.94–1.00)	0.94 (0.71–1.00)	1.00 (0.96–1.00)	0.97	0.96
	E2	0.94 (0.70–1.00)	0.99 (0.94–1.00)	0.94 (0.70–1.00)	0.99 (0.94–1.00)	0.94	0.93
	E3	1.00 (0.79–1.00)	0.99 (0.94–1.00)	0.94 (0.71–1.00)	1.00 (0.96–1.00)	0.97	0.96
	R3	0.94 (0.70–1.00)	0.99 (0.94–1.00)	0.94 (0.70–1.00)	0.99 (0.94–1.00)	0.94	0.93
	R2	0.94 (0.70–1.00)	0.98 (0.92–1.00)	0.88 (0.64–0.99)	0.99 (0.93–1.00)	0.91	0.89
	R1	0.94 (0.70–1.00)	0.99 (0.94–1.00)	0.94 (0.70–1.00)	0.99 (0.94–1.00)	0.94	0.93
	PA1	1.00 (0.79–1.00)	0.87 (0.78–0.93)	0.59 (0.39–0.78)	1.00 (0.95–1.00)	0.74	0.72
	PA2	1.00 (0.79–1.00)	0.87 (0.78–0.93)	0.59 (0.39–0.78)	1.00 (0.95–1.00)	0.74	0.72
	PA3	1.00 (0.79–1.00)	0.88 (0.79–0.94)	0.62 (0.41–0.80)	1.00 (0.95–1.00)	0.76	0.74

Medical staff performance was assessed during the DL algorithm–unassisted phase. Sensitivity, specificity, PPV, and NPV are presented with exact 95% confidence intervals; F1 score and MCC are presented as point estimates. DL, deep learning; PPV, positive predictive value; NPV, negative predictive value; MCC, Matthews correlation coefficient; F1, F1 score. E1–E3, emergency medicine specialists; R1–R3, emergency medicine residents (first to third year); PA1–PA3, physician assistant nurses.

**Table 3:** Agreement of the DL algorithm and medical staff members with the reference standard in the external validation dataset.

Medical Staff	Agreement, κ (95% CI)
DL algorithm	0.92 (0.84–0.98)
E1	0.96 (0.90–1.00)
E2	0.91 (0.82–0.98)
E3	0.98 (0.93–1.00)
R3	0.94 (0.87–1.00)
R2	0.85 (0.75–0.95)
R1	0.82 (0.71–0.92)
PA1	0.59 (0.46–0.72)
PA2	0.60 (0.47–0.73)
PA3	0.57 (0.44–0.70)

Medical staff performance was assessed during the DL algorithm–unassisted phase. Agreement is expressed as Cohen’s κ with bootstrap 95% CIs. DL, deep learning; E1–E3, emergency medicine specialists; R1–R3, emergency medicine residents (first to third year); PA1–PA3, physician assistant nurses; CI, confidence interval.

**Table 4:** Overall accuracy and lesion-specific balanced accuracy of nine medical staff members before DL algorithm assistance.

Medical Staff	Overall Accuracy	Urticaria BA (95% CI); *p*	Herpes Zoster BA (95% CI); *p*	Cellulitis BA (95% CI); *p*
E1	0.98	0.98 (0.96–1.00); 1.00	0.99 (0.98–1.00); 1.00	0.99 (0.98–1.00); 0.63
E2	0.95	0.97 (0.94–0.99); 1.00	0.97 (0.95–0.99); 0.69	0.96 (0.89–1.00); 1.00
E3	0.99	0.99 (0.97–1.00); 0.63	1.00 (1.00–1.00); 0.50	0.99 (0.98–1.00); 0.63
R3	0.97	0.97 (0.93–1.00); 1.00	0.99 (0.98–1.00); 1.00	0.96 (0.89–1.00); 1.00
R2	0.92	0.93 (0.88–0.97); 0.23	0.97 (0.94–0.99); 0.45	0.96 (0.88–1.00); 1.00
R1	0.90	0.92 (0.86–0.96); 0.09	0.95 (0.91–0.98); 0.11	0.96 (0.89–1.00); 1.00
PA1	0.74	0.81 (0.74–0.86); <0.001	0.88 (0.81–0.94); 0.001	0.94 (0.90–0.97); 0.06
PA2	0.75	0.80 (0.73–0.86); <0.001	0.89 (0.82–0.95); 0.004	0.94 (0.90–0.97); 0.06
PA3	0.73	0.78 (0.70–0.85); <0.001	0.86 (0.77–0.93); <0.001	0.94 (0.90–0.97); 0.07

Overall accuracy is the proportion of images correctly classified as one of the three disorders. BA is defined as (sensitivity + specificity)/2 and is presented with 95% CIs. *p*-values are from McNemar’s exact tests of paired image-level classifications between each medical staff member and the DL algorithm and do not directly test differences in balanced accuracy. CI, confidence interval; DL, deep learning; BA, balanced accuracy. E1–E3, emergency medicine specialists; R1–R3, emergency medicine residents (first to third year); PA1–PA3, physician assistant nurses.

**Table 5:** Overall accuracy and lesion-specific balanced accuracy of nine medical staff members after DL algorithm assistance.

Medical Staff	Overall Accuracy	Urticaria BA (95% CI); *p*	Herpes Zoster BA (95% CI); *p*	Cellulitis BA (95% CI); *p*
E1	1.00	1.00 (1.00–1.00); 0.25	1.00 (1.00–1.00); 0.50	1.00 (1.00–1.00); 0.25
E2	0.99	0.99 (0.97–1.00); 0.63	1.00 (1.00–1.00); 0.50	0.99 (0.98–1.00); 0.63
E3	1.00	1.00 (1.00–1.00); 0.25	1.00 (1.00–1.00); 0.50	1.00 (1.00–1.00); 0.25
R3	0.99	0.99 (0.97–1.00); 0.63	1.00 (1.00–1.00); 0.50	0.99 (0.98–1.00); 0.63
R2	0.99	0.99 (0.97–1.00); 0.63	1.00 (1.00–1.00); 0.50	0.99 (0.98–1.00); 0.63
R1	0.98	0.98 (0.96–1.00); 1.00	0.99 (0.98–1.00); 1.00	0.99 (0.98–1.00); 0.63
PA1	0.97	0.98 (0.96–1.00); 1.00	0.97 (0.91–1.00); 1.00	0.99 (0.97–1.00); 1.00
PA2	0.97	0.98 (0.95–1.00); 1.00	0.99 (0.98–1.00); 1.00	0.99 (0.97–1.00); 1.00
PA3	0.97	0.98 (0.95–1.00); 1.00	0.99 (0.97–1.00); 1.00	0.99 (0.98–1.00); 0.63

BA is defined as (sensitivity + specificity)/2 and is presented with 95% CIs. *p*-values are from McNemar’s exact test of paired image-level classifications between each medical staff member and the DL algorithm and do not directly test differences in balanced accuracy. CI, confidence interval; DL, deep learning; BA, balanced accuracy. E1–E3, emergency medicine specialists; R1–R3, emergency medicine residents (first to third year); PA1–PA3, physician assistant nurses.

## Data Availability

The data presented in this study are available upon reasonable request from the corresponding author. The data are not publicly available because of privacy restrictions on the clinical images. The trained model weights and inference code are not publicly available at this preliminary stage; their release will be expanded in the subsequent validation study.

## Supplementary Materials

The following supporting information can be downloaded at: https://www.mdpi.com/article/10.3390/jcm15218602/s1, Supplementary Table S1: Comparison of representative studies on deep learning for skin disease classification and the present study; Supplementary Table S2: Checklist for Artificial Intelligence in Medical Imaging (CLAIM), 2024 update; Supplementary Table S3: Sensitivity analyses for the effect of DL algorithm assistance in the mixed-effects model.

## Abbreviations

The following abbreviations are used in this manuscript:

AI	Artificial intelligence
BA	Balanced accuracy
CI	Confidence interval
CLAIM	Checklist for Artificial Intelligence in Medical Imaging
DL	Deep learning
ED	Emergency department
GEE	Generalized estimating equation
IRB	Institutional review board
MCC	Matthews correlation coefficient
NPV	Negative predictive value
PA	Physician assistant nurse
PACS	Picture archiving and communication system
PPV	Positive predictive value
AUC	Area under the receiver operating characteristic curve
ECE	Expected calibration error
Grad-CAM	Gradient-weighted class activation mapping
IQR	Interquartile range
OR	Odds ratio
ROC	Receiver operating characteristic
