# Supplementary material for: Classification Performance of a Deep Learning Algorithm for Common Skin Lesions in the Emergency Department: A Multicenter Preliminary Validation Study
Source: J Clin Med. 2026 Nov 4;15(21):8602. doi: 10.3390/jcm15218602 (PMC13764590; doi:10.3390/jcm15218602)
Supplement: Supplementary file 1 [file jcm-15-08602-s001.zip › jcm-4597042-supplementary.pdf]

Supplementary Materials

Classification Performance of a Deep Learning Algorithm for Common Skin Lesions in the Emergency Department: A Multicenter Preliminary Validation Study (jcm-4597042)

Table S1. Comparison of representative studies on deep learning for skin disease classification and the present study; Table S2. Checklist for Artificial Intelligence in Medical Imaging (CLAIM), 2024 update; Table S3. Sensitivity analyses for the effect of DL algorithm assistance on correct classification in the mixed-effects model.

Supplementary Table S1. Comparison of representative studies on deep learning for skin disease classification and the present study.

Study (year)	Data source and image type	Classification task	Validation	Comparison with clinicians	Assessment of algorithm assistance	Main findings
Esteva et al. (2017) [7]	129,450 images covering 2,032 skin diseases, including clinical and dermoscopic images, collected from repositories and Stanford Hospital	Binary tasks: keratinocyte carcinoma vs. benign seborrheic keratosis; melanoma vs. benign nevus (clinical and dermoscopic)	Biopsy-confirmed held-out test images	21 board-certified dermatologists	Not evaluated	CNN performance on par with dermatologists for binary skin cancer tasks
Fujisawa et al. (2019) [11]	4,867 clinical images from 1,842 patients with skin tumours, single university hospital (Japan)	14 diagnoses (skin tumours); also benign vs. malignant	Internal test set from the same institution; no external validation	13 board-certified dermatologists and 9 trainees	Not evaluated	14-class accuracy 76.5% (DCNN) vs. 59.7% (dermatologists) and 41.7% (trainees); benign/malignant accuracy 92.4% vs. 85.3% and 74.4%
Liu et al. (2020) [12]	Development data comprising 16,114 teledermatology cases from 17 primary care sites in the USA; clinical photographs and clinical information	Differential diagnosis of 26 common skin conditions (secondary output over 419 conditions)	Two validation sets from the same teledermatology service: set A (3,756 cases) and set B (963 cases); clinician comparison performed on set B	6 dermatologists, 6 primary care physicians, and 6 nurse practitioners	Not evaluated	Top-1 accuracy 0.66 (DLS) vs. 0.63 (dermatologists), 0.44 (PCPs), 0.40 (NPs)
Han et al. (2020) [13]	220,680 images covering 174 skin disorders	Multiclass classification of 134 skin disorders, malignancy, and treatment options	Edinburgh dataset (1,300 images; 10 disorders) and SNU dataset (2,201 images; 134 disorders)	21 dermatologists, 26 dermatology residents, and 23 non-medical participants in the malignancy assessment	Evaluated: participants revised answers after seeing the algorithm output	Malignancy sensitivity improved from 77.4% to 86.8% among dermatologists and residents and from 47.6% to 87.5% among non-medical participants with algorithm output
Jain et al. (2021) [14]	1,048 retrospective teledermatology cases (consumer-camera or mobile photographs plus history) covering 120 skin conditions (USA)	AI-assisted differential diagnosis of skin conditions	Cases from the validation sets of the same teledermatology service	20 PCPs and 20 NPs; dermatologist panel as reference	Randomized case batches assessed with or without AI assistance; no same-case crossover within an individual clinician	Top-1 agreement with the dermatologist reference increased from 48% to 58% (PCPs) and from 46% to 58% (NPs) with AI assistance

Study (year)	Data source and image type	Classification task	Validation	Comparison with clinicians	Assessment of algorithm assistance	Main findings
Escalé-Besa et al. (2023) [25]	100 consecutive adults with a skin problem at six primary care centres (Spain); clinical photographs taken by general practitioners (dermoscopic images in 52%)	Ranked differential diagnosis (commercial tool covering 44 skin diseases; top-1/top-3/top-5)	Prospective real-world evaluation of an existing tool; 18 cases had diagnoses outside the tool's scope	11 general practitioners (GPs) and teledermatologists; dermatologist consensus as reference	Only self-reported usefulness (GPs diagnosed first, then viewed the AI output); accuracy with assistance not measured	Top-1 accuracy was 39% for the algorithm, 64% for general practitioners, and 72% for dermatologists
Present study	6,000 non-dermoscopic clinical images from 4,815 patients at two emergency medical centres in Korea, acquired during routine care	Three-class classification of urticaria, herpes zoster, and cellulitis	Independent external validation set (100 images, 100 patients) from a third emergency medical centre not involved in development	9 ED medical staff members: 3 emergency medicine specialists, 3 residents, and 3 PA nurses	Evaluated: sequential design, unassisted phase followed by assisted phase after a 15-day washout, same 100 images	External accuracy was 0.96 and macro-average AUC was 0.98. The assisted phase was associated with higher odds of correct classification (OR 25.9, 95% CI 11.7–57.2). Mean pairwise Cohen's κ increased from 0.72 to 0.97.

Abbreviations: AI, artificial intelligence; AUC, area under the receiver operating characteristic curve; CI, confidence interval; CNN, convolutional neural network; DCNN, deep convolutional neural network; DLS, deep learning system; ED, emergency department; GP, general practitioner; NP, nurse practitioner; OR, odds ratio; PA, physician assistant nurse; PCP, primary care physician; SNU, Seoul National University.

Notes: Studies were selected to illustrate skin cancer classification, multiclass classification of skin conditions, and assessment of algorithm assistance. This is a representative rather than exhaustive review. Numerical results are reported as presented in the cited publications. Performance estimates are not directly comparable across studies because the target conditions, datasets, reference standards, and evaluation designs differ. For the present study, mean pairwise Cohen's κ describes agreement between pairs of medical staff members and is distinct from Fleiss' κ reported elsewhere in the manuscript.

Reference numbers in brackets refer to the reference list of the main text.

Supplementary Table S2. Checklist for Artificial Intelligence in Medical Imaging (CLAIM), 2024 update.

Item numbering follows the CLAIM 2024 update (Tejani AS, et al. Radiol Artif Intell. 2024;6(4):e240300). "Location" indicates the section of the revised manuscript in which the item is addressed, or the reason an item is not applicable or not reported.

Section	Item	Item description	Location in manuscript / note
Title/Abstract	1	Identification as a study of AI methodology, specifying the category of technology used (e.g., deep learning)	Title; Abstract
Title/Abstract	2	Structured summary of study design, methods, results, and conclusions	Abstract (Background/Objectives, Methods, Results, Conclusions)
Introduction	3	Scientific and/or clinical background, including the intended use and role of the AI approach	Introduction
Introduction	4	Study aims, objectives, and hypotheses	Introduction, final paragraph
Methods: Study design	5	Prospective or retrospective study	Methods 2.1 (multicenter, retrospective)
Methods: Study design	6	Study goal (e.g., model creation, exploratory study, feasibility study)	Methods 2.1 (preliminary validation study)
Methods: Data	7	Data sources	Methods 2.1–2.3 (PACS and electronic medical records of three emergency medical centers)
Methods: Data	8	Inclusion and exclusion criteria	Methods 2.3; Figure 1
Methods: Data	9	Data preprocessing steps	Methods 2.3 and 2.4 (square crop centred on the lesion; resizing to 224×224 pixels, RGB, aspect ratio preserved)
Methods: Data	10	Selection of data subsets, if applicable	Methods 2.4 (training, internal validation, and external validation sets)
Methods: Data	11	De-identification methods	Methods 2.3
Methods: Data	12	How missing data were handled	Not applicable: image-only inputs; patients whose final diagnosis could not be confirmed were excluded (Methods 2.4; Figure 1)
Methods: Data	13	Image acquisition protocol	Methods 2.3 (personal smartphone cameras under ambient lighting; not standardized)
Methods: Reference standard	14	Definition of method(s) used to obtain the reference standard	Methods 2.4 (final diagnosis established by a dermatologist and recorded in the electronic medical record, based on the full clinical context; for cellulitis, clinical course and treatment response)
Methods: Reference standard	15	Rationale for choosing the reference standard	Methods 2.4 (dermatologist diagnosis from the full clinical context rather than the photograph alone; no definitive laboratory standard for cellulitis)
Methods: Reference standard	16	Source of reference standard annotations; qualifications of annotators	Methods 2.4 (board-certified dermatologists; diagnosis recorded before model development)
Methods: Reference standard	17	Annotation of test set	Methods 2.4 (same reference standard applied to the external validation set)
Methods: Reference standard	18	Measures of inter- and intrarater variability of annotators	Not reported: a single documented dermatologist diagnosis was used; no independent re-annotation was performed (Limitations)
Methods: Data partitions	19	How data were assigned to partitions; proportions	Methods 2.4 (random patient-level 9:1 split, not stratified by class)

Methods: Data partitions	20	Level at which partitions are disjoint	Methods 2.4 (patient level; external validation set from a separate institution)
Methods: Testing data	21	Intended sample size and how it was determined	Methods 2.7 (no prior sample-size calculation); Limitations
Methods: Model	22	Detailed description of model, including inputs, outputs, and layers	Methods 2.4 and 2.5 (ResNet-50; 224×224×3 input; three-class softmax output)
Methods: Model	23	Software libraries, frameworks, and packages	Methods 2.4 and 2.7 (TensorFlow 2.17, Google Colaboratory; NumPy, scikit-learn; R 4.3.3, lme4 1.1-35)
Methods: Model	24	Initialization of model parameters (e.g., randomization, transfer learning)	Methods 2.4 (ImageNet-pretrained weights; fixed random seed 42)
Methods: Training	25	Details of training approach, including augmentation, hyperparameters, and number of models trained	Methods 2.4 (Adam optimizer; learning rate 0.0001; batch size 16; up to 100 epochs; categorical cross-entropy; no augmentation or class weighting)
Methods: Training	26	Method of selecting the final model	Methods 2.4 (checkpoint with the lowest internal validation loss)
Methods: Training	27	Ensembling technique, if applicable	Not applicable: single model
Methods: Evaluation	28	Metrics of model performance	Methods 2.7 (accuracy, sensitivity, specificity, balanced accuracy, MCC, Cohen's κ, AUC, Brier score, ECE)
Methods: Evaluation	29	Statistical measures of significance and uncertainty	Methods 2.7 (Clopper–Pearson, bootstrap, and Wilson 95% CIs; mixed-effects logistic regression)
Methods: Evaluation	30	Robustness or sensitivity analysis	Methods 2.7; Supplementary Table S3; confidence-threshold analysis (Figure 2C)
Methods: Evaluation	31	Methods for explainability or interpretability	Methods 2.5 (Grad-CAM, final paragraph); Figure 3
Methods: Evaluation	32	Evaluation on internal data	Methods 2.4 (internal validation set used for model selection only; development-stage accuracy stated)
Methods: Evaluation	33	Testing on external data	Methods 2.4; Results 3.3 (independent third institution, n = 100)
Methods: Evaluation	34	Clinical trial registration	Not applicable: not a clinical trial
Results: Data	35	Numbers of patients or examinations included and excluded	Figure 1; Results 3.1
Results: Data	36	Demographic and clinical characteristics of cases in each partition and dataset	Table 1; Results 3.1; lesion site of external set in Results 3.3
Results: Model performance	37	Performance metrics and measures of statistical uncertainty	Results 3.3; Table 2; Figure 2
Results: Model performance	38	Estimates of diagnostic performance and their precision	Results 3.3; Table 2 (95% CIs)
Results: Model performance	39	Failure analysis of incorrect results	Results 3.3; Figure 3 (all four misclassified images with Grad-CAM)
Discussion	40	Study limitations, including potential bias, statistical uncertainty, and generalizability	Discussion, Limitations
Discussion	41	Implications for practice, including intended use and/or clinical role	Discussion (decision support for medical staff; not a stand-alone triage tool)
Other information	42	Reference to the full study protocol or additional technical details	Not available: no separate protocol; technical details are given in Methods 2.4
Other information	43	Statement about availability of software, trained model, and/or data	Data Availability Statement
Other information	44	Sources of funding and other support; role of funders	Funding

Supplementary Table S3. Sensitivity analyses for the effect of DL algorithm assistance in the mixed-effects model.

Model	Specification	OR for assistance	95% CI	p
S0	Primary model: random intercepts for medical staff and image; fixed effects assistance + role	25.9	11.7–57.2	<0.001
S1	Staff random effect dropped (image random effect only)	25.9	11.7–57.2	<0.001
S2	Role fixed effect dropped (staff variance estimated; SD 1.65)	26.3	11.8–58.2	<0.001
S3	Four algorithm-misclassified images excluded (n = 96 images)	25.7	11.6–56.9	<0.001
S4	Specialists excluded (residents and PA nurses)	23.8	10.7–53.2	<0.001
S5	PA nurses only	23.5	9.6–57.7	<0.001
S6	Primary model, parametric bootstrap 95% CI (500 replicates)	25.9	13.0–68.1	—
S7	Generalized estimating equation, exchangeable correlation within image (population-averaged OR)	10.6	4.0–27.9	<0.001

Outcome: correct classification of each image (1,800 readings: 100 images × 9 medical staff × 2 phases). Fixed effects: DL algorithm assistance (assisted vs. unassisted) and staff role (specialist, resident, PA nurse); S2 and S5 omit the role term. Random intercepts: medical staff and image (crossed) unless stated otherwise. Models fitted by maximum likelihood with Laplace approximation (R 4.3.3, lme4 1.1-35); p-values from Wald tests. In the primary model the medical-staff random-effect variance was estimated at zero (boundary); the image random-effect SD was 2.05 (latent-scale intraclass correlation 0.56). The assistance-by-role interaction was not significant (likelihood-ratio p = 0.60). The GEE estimate (S7) is a population-averaged odds ratio and is expected to be smaller than the conditional odds ratios of the mixed-effects models given the large image-level intraclass correlation. CI, confidence interval; DL, deep learning; GEE, generalized estimating equation; OR, odds ratio; PA, physician assistant; SD, standard deviation.
